# Personalized causal explanations of a robot’s behavior

**DOI:** 10.3389/frobt.2025.1637574

**Published:** 2025-10-08

**Authors:** José Galeas, Suna Bensch, Thomas Hellström, Antonio Bandera

**Affiliations:** 1 Department Tecnología Electrónica, University of Málaga, Málaga, Spain; 2 Department Computing Science, University of Umeå, Umeå, Sweden

**Keywords:** explainable robots, understandable robots, personalized explanations, speaker role recognition, human–robot interaction, causal explanations

## Abstract

The deployment of robots in environments shared with humans implies that they must be able to justify or explain their behavior to nonexpert users when the user, or the situation itself, requires it. We propose a framework for robots to generate personalized explanations of their behavior by integrating cause-and-effect structures, social roles, and natural language queries. Robot events are stored as cause–effect pairs in a causal log. Given a human natural language query, the system uses machine learning to identify the matching cause-and-effect entry in the causal log and determine the social role of the inquirer. An initial explanation is generated and is then further refined by a large language model (LLM) to produce linguistically diverse responses tailored to the social role and the query. This approach maintains causal and factual accuracy while providing language variation in the generated explanations. Qualitative and quantitative experiments show that combining the causal information with the social role and the query when generating the explanations yields the most appreciated explanations.

## 1 Introduction

Making robots understandable is generally acknowledged as important for improving safety, user experience, trust, and efficiency (Hellström and Bensch, 2018). Understandable robots may, for example, verbally explain their actions and decisions as a response to questions asked by interacting humans. Such explainability is especially important in sensitive settings, such as eldercare or medical assistance, where lack of information or clarity could result in physical or psychological harm.

Explainability and causality are closely intertwined concepts (Setchi et al., 2020). Lewis, in his seminal work, described explaining an event as “providing information about its causal history” (Lewis, 1986). Earlier related work (Lindner and Olz, 2022; Chakraborti et al., 2017) often shares this focus on actions, effects, and their technical causes, that is, on *what* to explain.

However, an explanation solely based on cause and effect reasoning might not be sufficient if the robot interacts with humans with diverse backgrounds. For example, in an eldercare home, humans interacting with an assistive robot may be residents, medical staff, family members, or technicians. We build on several observations.

First, not all inquirers have the same wishes and needs for information. For example, a family member and a nurse asking “Why did Maria choose to eat meat today?” may want different aspects included in an explanation. The *social role* of the user also influences which words and expressions are appropriate to use. Second, the tone and wordings of a query may express an intentional or latent wish to have certain aspects addressed in the causal explanation. For example, a family member asking “Why did my father Alberto have meat today?” or “Why did my father Alberto have meat today again?” may reflect a wish for different aspects to be included in the explanation. To address the two observations above, we take the social role of the person asking the question and the question itself into account when generating explanations, thereby addressing the additional focus on *how* to explain.

In this paper, we present a framework for robots to generate personalized explanations for events when a human requests an explanation. Our approach maintains factual and causal correctness, which are crucial for any robotic application, while leveraging a large language model (LLM) to personalize and diversify the language of the explanations. Providing linguistic variation of the explanations is important for humans who interact with the robot on a regular basis to avoid monotony and reduced engagement.

More specifically, robot events (e.g., actions and tasks) are stored in a causal log where they are structured into cause and effect pairs. Given a human query q , we use machine learning to extract the robot event e for which an explanation is requested and to identify the likely social role s of the human asking. For the identified robot event, the corresponding cause and effect in the causal log are processed to provide an initial explanation. This initial explanation is then refined by an LLM (Llama) to produce linguistically varied explanations that are tailored to the social role and the actual query. The initial explanation that is based on the data from the causal log maintains factual correctness, whereas the LLM adds language variation.

To evaluate our approach, three quantitative and qualitative experiments were performed. Experiment I investigated the effects of including combinations of cause and effect, social role, and query when generating an explanation. Experts and 30 participants assessed the quality of the generated explanations, and a statistical analysis of the results indicates that combining cause and effect with the human’s social role and the original query yields the most preferred explanations. The findings further show that the second most preferred option includes cause-and-effect structures and social roles (compared to cause-and-effect and query combinations). This indicates that incorporating the social role in causal explanations plays a significant role. Experiment II verifies that the social role of the enquirer is identified with high accuracy, and Experiment III shows a high semantic similarity between the system-generated explanations and human-generated explanations that served as ground truth. The paper is organized as follows. Section 2 consists of Section 2.1 that provides an overview of related work and Section 2.2 in which basic terms and concepts used in the paper are introduced. Section 3 describes the proposed methodology for the generation of explanations, followed by a description of experiments and results in Section 4. Section 5 discusses challenges and limitations, and Section 6 finalizes the paper with conclusions and ideas for future work.

## 2 Technical background

### 2.1 Related work

#### 2.1.1 What is an explanation?

Humans have an innate tendency to construct explanations, a process crucial for understanding and making sense of the world around us. Explanations help build the foundation for reasoning and generalization (Lombrozo, 2006). According to Federer et al. (2015) and Meyer and Schnell (2020), explanations are composed of two key elements: the explanandum, the phenomenon being explained, and the explanation itself, which provides the rationale or reasoning. On a broader level, Norris et al. (2005) defined an explanation as an “act intended to make something clear, understandable, or intelligible” (p. 546). For explanations to be effective, they must be meaningful and adapted to the abilities and needs of the audience (Stefani and Tsaparlis, 2009). Similarly, Tania Lombrozo characterized explanations as bridging the gap that enables others to comprehend an event (Lombrozo, 2006). This process is inherently cognitive, involving answers to questions—often framed as “why”—that are typically constrained by context. The recipient of an explanation is usually less interested in the mere occurrence of an event and more focused on understanding why it happened in a particular instance rather than in alternative, counterfactual scenarios (Matarese et al., 2021).

The idea of crafting a universal theory of “good explanations” has been explored but remains unresolved due to the distinct needs of different disciplines (Pitt, 2009). For example, engineering often requires professionals to clearly communicate their decisions and solutions, as part of their nontechnical competencies (Kaplar et al., 2021). Reverse engineering, in particular, focuses on understanding “how existing artifacts produce their overall functions in terms of underlying mechanisms” (van Eck, 2015).

From a technological point of view, Pitt (2009) advocated for a theory of technological explanations to clarify the purpose and functionality of artifacts. He argued that such explanations must reference the broader system in which a tool operates, as its design, function, or structure … “can only be adequately explained by reference to the system” (p. 861). Although scientific explanations aim to reveal “why the world works the way it does” in specific contexts (p. 862), technological explanations address practical questions such as “How does this work?” or “Why does the artifact do this?”.

#### 2.1.2 Explanations in HRI

In human–robot interaction (HRI), the ability to provide clear and meaningful explanations is essential for the widespread acceptance of robots in critical tasks (Edmonds et al., 2019). Studies, such as those by Sakai and Nagai (2022) and Chakraborti et al. (2021), highlight that true communication between humans and robots requires more than a basic understanding of commands or questions. Robots must also be capable of recognizing and interpreting a person’s internal state while conveying their own reasoning in ways that humans can easily grasp (Zakershahrak and Ghodratnama, 2020). For a robot to anticipate the needs of a person or adapt its behavior effectively, it must possess mechanisms to infer human intentions and provide clear and actionable insights into its own actions. This capability not only helps robots align with human expectations but also builds trust. Robots equipped with these abilities are referred to as explainable autonomous robots (XARs) (Stange et al., 2022; Sakai and Nagai, 2022). This concept parallels explainable artificial intelligence (XAI), but with a critical distinction. Whereas XAI aims to enhance understanding and control by offering transparent justifications for decisions often centered on data-driven processes (Gjærum et al., 2023; Adadi and Berrada, 2018), XARs are primarily concerned with explaining their autonomous behaviors in a shared, dynamic environment. As Sakai and Nagai emphasized, this involves a shift from data-focused explainability to goal-driven explainability, where the robot must clearly articulate the rationale behind its actions in pursuit of its objectives (Sakai and Nagai, 2022; Zakershahrak and Ghodratnama, 2020). This focus is crucial for fostering effective collaboration and trust in human–robot partnerships. “Understandability” is suggested as a broader term than explainability (Hellström and Bensch, 2018) and covers not only a robot’s actions but also entities such as intentions, desires, knowledge, beliefs, emotions, perceptions, capabilities, and limitations of the robot. Furthermore, understandability may be achieved not only by uttering verbal explanations but also by other modalities and even by the robot’s actual motions and actions.

#### 2.1.3 Speaker role recognition

Typically framed in the process of determining the speaker’s turn in a homogeneous speech segment, speaker role recognition (SRR) seeks to determine the role of a speaker, considering that this role is characterized by the task performed by the speaker and the goals related to it (Flemotomos et al., 2019). Obviously, to solve the SRR problem, patterns must be identified to differentiate these roles. Different proposals have looked for these patterns at the low level, either in audio files or in rhythm and sound (e.g., the interviewer will use more interrogative words than the interviewee). Other authors agreed that language usually incorporates more information to solve this problem (Flemotomos et al., 2019; Prasad et al., 2022; Zuluaga-Gomez et al., 2023), so the aim is to exploit lexical variability to differentiate roles. Both sets of acoustic and lexical patterns can be used together, in approaches that combine automatic speech recognition (ASR) and SRR (Blatt et al., 2024). In any case, in recent years, traditional solutions have been replaced by deep learning.

Earlier work is built upon and advanced in this paper by integrating the enquirer and their mental state (through their social role) and the natural language query into the generation of more personalized explanations while maintaining causal correctness of the description of the robot’s behavior. As our experiments show, this approach results in explanations that are more appreciated by the enquirer and, in turn, make the robot more understandable and valuable to the interacting human.

### 2.2 Formalism and terminology

In this section, we introduce the terminology that will be used throughout the paper.

#### 2.2.1 Literals, robot event, and causal robot event

Starting with the basic entities the robot operates on, we define a *literal* as follows: Definition 1 [Literal] A Literal is a placeholder for a physical or virtual entity that the robot considers in its operation. Notation: *literal_name* or *literal_name1(literal_name2)*.

Literals are frequently used in the program code controlling the robot and are necessary when describing the operation of the robot. Examples of literals are as follows: 1, *Jose*, *Menu(Maria)*, *No_of_choices*, *Full*, and *Empty*. The functional notation *literal_name1(literal_name2)* should be interpreted as a specification of *literal_name1*. For example, *Menu(Maria)* refers to the specific menu that is connected to *Maria*. Moving on to entities that the robot may be asked to explain, we consider three categories of basic robot operations:

• Sensing or perception (e.g., the robot detects an object, a person, or low battery level).

• Cognition (e.g., the robot estimates the distance to an object or associates a value with a literal).

• Acting (e.g., the robot moves to a certain location or asks a person about their food preferences).

Based on these categories, we define a *robot event* as follows:

Definition 1 (Robot event). *A robot event is a predicate (i.e., a Boolean expression that evaluates to True or False) with zero or more arguments and represents a specific robot operation. The arguments* $arg_{i}$ *are literals. Notation: event_name(* $arg_{1}$ , …, $arg_{k}$ ), k≥0 .

For example, the one-argument event *Start_move_to_safe_distance(Jose)* represents the action where the robot moves away from the person identified as *Jose*, and the two-argument event *Assign(No_of_choices, 3)* represents the robot’s cognitive operation of associating the value 3 with the literal *No_of_choices*. As an alternative notation, this event may also be denoted as *No_of_choices = 3*. Similarly, the event *Assign(Menu(Maria), Full)* may also be denoted by *Menu(Maria) = Full*.

To describe the reasons why events occur, we introduce the notion of a *Causal robot event*, defined as follows:

Definition 2 (Causal robot event). *A causal robot event comprises a timestamped rule:*

$$t\left[\alpha_{1},\alpha_{2},\ldots ,\alpha_{k}\right]\to \beta ,$$

where t is the timestamp and the antecedent $\left[\alpha_{1},\alpha_{2},\ldots ,\alpha_{k}\right]$ is a list of robot events that were all True at time t, which caused the consequent robot event β to happen. The antecedent is referred to as the Cause and is sometimes denoted by α as a short notation. The consequent is referred to as the Effect.

Two examples of causal robot events are as follows:

• *Effect* β : “Use_case_menu_started (Jose)”
     *Causes* $\alpha_{1},\alpha_{2},\alpha_{3}$ : (“Person_detected (Jose),” “Menu (Jose) = False,” “Therapy_time = False”)
     timestamp t:123213123

     At time t , all events in *Cause* were True, and the *Effect* occurred.

• *Effect* β : “Person_detected (Jose)”
     *Cause* α : ()
     timestamp t:123213923

     The *Effect* occurred at time t without any specified prior conditions to hold true; that is, a person was detected independently of events internal to the robot.

It should be noted that for causal events with non-empty causes, the “causation” reflects how the software controlling the robot is written: certain conditions (the Cause) lead the program to follow a path where Effect is performed. The only exception is Effects related to perception, which depend on external conditions, and the Cause is, in these cases, an empty list.

#### 2.2.2 Causal log and dictionary

The causal log is a component of a complex cognitive robot architecture CORTEX (Galeas et al., 2025). It is a tabular high-level episodic memory representation, and the causal log entries are automatically generated in real-time settings. In particular, the cause column is filled in with states/actions extracted from behavior trees that control the robot’s behavior. These behavior trees are defined at design time and specify both task steps and conditions that trigger robot behavior changes. When such a change occurs, the system automatically records one row in the causal log.

Formally, all occurrences of causal events are recorded in the *causal log*, which is defined as follows:

Definition 3 (Causal log). *A causal log is a table with numbered rows. Three columns represent causal events: the timestamp* t *, the effect* β *, and the cause* $\alpha_{1},\ldots ,\alpha_{k}$ *. The additional column cause_idx contains a list (possibly empty) of links that connect each* $\alpha_{i}$ *with a prior occurrence of a causal event with the effect equal to* $\alpha_{i}$ *. More precisely, the cause_idx at row* j *is a list* $\left(r_{1},\ldots ,r_{k}\right)$ *of row numbers in the causal log, where each* $r_{i}$ *belongs to condition* $\alpha_{i}$ *in the cause at row* j . $r_{i}$ *is set to the largest row number less than* j *, for which the effect column equals* $\alpha_{i}$ .

An example of a causal log is given in Table 1.

**TABLE 1:** The robot records all occurrences of causal events in the causal log, which is represented as a table.

	Timestamp t	Effect β	Cause $\left(\alpha_{1},\ldots ,\alpha_{k}\right)$	cause_idx
1	1727677625	Menu (Jose) = Empty	( )	…
2	1727678125	Use_case_wandering_started	( )	
3	1727681232	Menu (Alberto) = Empty	( )	…
4	1727679180	Neuron (Alberto) = True	( )	
5	1727679030	Ordered_food (Maria)	(Use_case_menu_ended (Maria))	
6	…	…	…	…
7	1727679060	Therapy_time = False	( )	
8	1727679070	Person_detected (Maria)	( )	
9	1727679090	Menu (Maria) = Full	(Ordered_food (Maria))	(5)
10	…	…	…	…
11	1727679140	Person_detected (Jose)	( )	
12	…	…	…	
13	1727699200	Use_case_menu_started (Jose)	(Person_detected (Jose), Menu (Jose) = Empty, Therapy_time = False)	(11,1,7)
14	1727679120	Therapy_time = True	( )	
15	1727679140	Person_detected (Alberto)	( )	
16	1727679180	…	…	
17	1727699200	Use_case_cognitive_started (Alberto)	(Person_detected (Alberto), Neuron (Alberto) = True, Therapy_time = True)	(15,4,14)
18	1727679180	Neuron (Alberto) = False	(Use_case_cognitive_started (Alberto))	(17)
19	1727699200	Use_case_menu_started (Alberto)	(Person_detected (Alberto), Menu (Alberto) = Empty, Neuron (Alberto) = False)	(15,3,18)

The causal log contains causal chains or causal dependencies of linked causal events.

The generation of explanations requires descriptions of all events that may occur in the causal log. Such descriptions are provided in a *dictionary*, as exemplified in Table 2. Parameterized event names $e_{p}$ and descriptions $des_{p}$ reduce the size of the dictionary considerably. Parameters are instantiated when the dictionary is used to find initial basic descriptions for robot events in the causal log that should be explained. A *dictionary* is formally defined as follows:

**TABLE 2:** The dictionary provides basic descriptions $des_{p}$ for events $e_{p}$ .

Parameterized event name $e_{p}$	Parameterized event description $des_{p}$
Start_move_to_safe_distance_from (&1)	The robot started to move to reach a safe distance from &1
Person_detected (&1)	Person &1 was detected by the robot’s facial recognition camera
Menu (&1) = Empty	&1 had not ordered any food today
Menu (&1) = Full	&1 had already ordered food today
Therapy_time = True	It was the right time for cognitive therapy
Neuron (&1) = True	Person &1 was scheduled for the cognitive therapy today
Use_case_reminder_started (&1)	The robot reminded &1 about an activity in their agenda
Use_case_cognitive_started (&1)	When the robot recognized &1 who participates in the therapy, it offered the possibility to start an activity which is conducted using the robot’s touch screen

Both $des_{p}$ and $e_{p}$ may contain variables that are instantiated when explanations are generated.

Definition 4 (Dictionary). *A dictionary is a table where all possible events are listed in separate rows. The table has two columns: parameterized event* $e_{p}$ *and parameterized event description* $des_{p}$ *. Both columns may contain variables* & n *that are substituted for actual values when explanations are generated.*

#### 2.2.3 Questions

A person interacting with a robot may ask the robot several types of questions. In this paper, we restrict the scope to questions about the *reasons for robot events* that appear in the robot’s causal log. Hence, the robot can answer questions related to what it has perceived (sensing), “thought about” (cognition), and done (acting). Examples of such questions are “Why are you asking me about physio therapy?,” “How come you returned to the charging station?,” and “Why are you moving around in the living room?”.

#### 2.2.4 Causal explanation

A question may be answered with a *causal explanation*. A *causal explanation* for an effect β in a causal event appearing in a causal log is a text string describing the direct and indirect causes of β . The direct causes $\alpha_{1},\ldots ,\alpha_{k}$ are given in the corresponding column in the causal log. The indirect causes of β can be found by, for each $\alpha_{i}$ , looking up rows in the causal log where the effect column matches $\alpha_{i}$ . For this, the row numbers in the *cause_idx* column are utilized. Each cause of $\alpha_{i}$ is an indirect cause of β . This can, in principle, continue recursively until all causes are empty—corresponding to events that cannot be explained by referring to other events in the causal log, but rather to external conditions (e.g., related to perception). Although an explanation may be observed as more “accurate” if it also refers to indirect causes, it may become overly complex and difficult to understand. The optimal trade-off between complexity and understandability depends on factors such as the purpose of the explanation, the user’s ability to understand detailed and complex information, and the time available to communicate the explanation. In this paper, we only consider direct causes when constructing explanations. However, the methodology presented can also handle the recursive inclusion of indirect causes.

## 3 Methodology

### 3.1 Methodological overview

**FIGURE 1:**
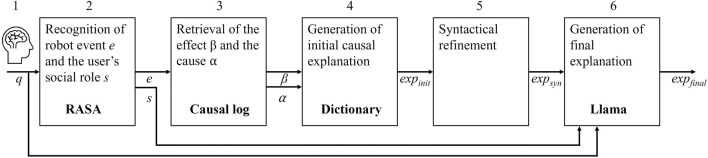
Methodological overview illustrating the process of generating personalized causal explanations based on user queries, social roles, and cause-and-effect structures.

Figure 1 shows an overview of the methodology that generates a final explanation $exp_{\mathrm{final}}$ in response to a user’s question q . It comprises the following six steps:

1. Input of a natural language query q from a user.
2. Recognition of a robot event e and the user’s social role s from q . This is done using the intent recognition component in Rasa, trained using supervised learning, as described in Section 3.2.
3. Retrieval of the effect β and the cause α from the causal log, as described in Section 3.3.
4. Generation of initial causal explanation $exp_{\mathrm{init}}$ based on descriptions in the dictionary for β and α (see Section 3.4).
5. Syntactical refinement of the initial explanation $exp_{\mathrm{init}}$ , resulting in a grammatically structured explanation $exp_{\mathrm{syn}}$ (see Section 3.4).
6. Generation of final explanation $exp_{\mathrm{final}}$ by prompting an LLM with a combination of the three factors $exp_{\mathrm{syn}}$ , s , and q (see Section 3.5).

An example of the inputs and outputs in each step of the process is given in Table 3. In the last step, all the three factors $exp_{\mathrm{syn}}$ , s , and q were used to generate the final explanation $exp_{\mathrm{final}}$ .

**TABLE 3:** Examples of inputs and outputs in each step of the process illustrated in Figure 1.

q	Why did my father, Alberto, have cognitive therapy today?
e	Use_case_cognitive_started (&1)
s	Family
β	Use_case_cognitive_started (Alberto)
α	Person_detected (Alberto), Neuron (Alberto) = True, Therapy time = True
$exp_{\mathrm{init}}$	Cause: person Alberto was detected by the robot’s facial recognition camera; person Alberto was scheduled for the cognitive therapy activity today; it was the right time for cognitive therapy. Effect: when the robot recognized Alberto who participates in the therapy, it offered the possibility to start an activity, which is conducted using the robot’s touch screen
$exp_{\mathrm{syn}}$	Because Alberto was detected by the robot’s facial recognition camera, Alberto was scheduled for the cognitive therapy activity today, and it was the right time for cognitive therapy. As a result, when the robot recognized Alberto who participates in the therapy, it offered the possibility to start an activity, which is conducted using the robot’s touch screen
$exp_{\mathrm{final}}$	I gave your father cognitive therapy because I recognized him and knew he had a scheduled session today, so I offered him the activity on my screen

The following subsections provide detailed descriptions of the steps in the process.

### 3.2 Recognition of the robot event and social role

In this step, the user’s natural language query q is mapped to a parameterized robot event e and a social role s related to the user. We utilized Rasa’s intent recognition component, which is intended to find mappings from natural language utterances to the speaker’s intent. For example, the intents behind “Good morning” and “What do you mean?” may be “greeting” and “clarification,” respectively. This mapping is created through supervised learning by providing pairs of utterances and intents. We used the same learning functionality but provided pairs of queries q and parameterized robot events $e_{p}$ (see Definition 2.2.2), such as the query “Why did my father, Alberto, have cognitive therapy today?” and the parameterized event “Use_case_cognitive_started (&1).” The model for robot event recognition was trained on 25 parameterized events $e_{p}$ , each associated with 20 query–event pairs $\left(q_{p,i},e_{p}\right)$ , 1≤i≤20 , resulting in a total of 500 training pairs. Half of the queries $q_{p,i}$ were collected from real users (e.g., residents, family members, and technical staff) during a previous project where a robot was deployed in a retirement home in Malaga, Spain (Jerez et al., 2024). This ensured that the queries reflected the actual concerns and needs of the target user groups. The other half of the queries $q_{p,i}$ were linguistic variations of the real users’ queries, generated using ChatGPT. Appropriate event templates from the dictionary were manually matched with each query. Examples of the robot event recognition are shown in Table 4, where system output for four use cases are shown: choosing from the menu, cognitive therapy, robot moving around in common room (i.e., wandering), and interaction with a human.

**TABLE 4:** Examples of robot event recognition.

Example 1. Robot event: use _ case _ menu	Example 2. Robot event: use _ case _ cognitive therapy
*“Why did my father Alberto choose his menu?”* Use_case_menu_started (&1), Confidence: 0.81 Use_case_menu_finished (&1), Confidence: 0.06 Menu_choices (&1), Confidence: 0.03 Person_finish_interact (&1), Confidence: 0.03 Person_interacting (&1), Confidence: 0.02 Looking_for_therapist (&1), Confidence: 0.01	*“Why did my father Alberto undergo cognitive therapy?”* Use_case_cognitive_started (&1), Confidence: 0.87 Use_case_cognitive_finished, Confidence: 0.03 Person_interacting (&1), Confidence: 0.02 Use_case_reminder_finished, Confidence: 0.02 Person_finish_interact (&1), Confidence: 0.01

Example 3. Robot event: use _ case _ wandering	Example 4. Robot event: use _ case _ person interaction
*“Why did you move around the living room?”* Use_case_wandering_started, Confidence: 0.87 Use_case_music_finished, Confidence: 0.08 Use_case_wandering_finished, Confidence: 0.02 Use_case_request_finished, Confidence: 0.01	*“Why were you interacting with Pedro?”* Person_interacting (&1), Confidence: 0.91 Person_finish_interact (&1), Confidence: 0.06 Looking_for_therapist (&1), Confidence: 0.01 Robot.activities = &1, Confidence: 0.00

In Example 4, the user query “Why were you interacting with Pedro?” is associated with the parameterized robot event “Person_interacting (&1)” with confidence 0.91.

Similarly, for social role recognition, we provided pairs of queries q and social roles s as training data. We considered the three social roles: *technician*, *resident*, and *family member*. The social role of a technician refers to a professional who is responsible for the maintenance of the social robot used in, for example, a retirement home. Technicians engage on a technical level with the robot, and robot explanations tailored to technicians should be focused on technology-oriented aspects. The social role of a resident refers to an independent older adult who resides in a retirement home and interacts with the robot. The interaction between a resident and social robot focuses on aspects that influence their daily lives (e.g., choosing lunch, cognitive therapy, and playing games in the common room.). Explanations for the resident should not be technical, but rather clarifying, easily understandable, accurate, and adaptive to the needs and preferences of the resident. The social role of a family member refers to visitors to the retirement home (e.g., children, grandchildren, relatives, and friends). The explanations to family members should also be clear, correct, and potentially more personal. Social roles guide how humans interact with each other and affect both what we talk about and how we talk (Eastman, 1985). Related to explanations, it is reasonable to assume that the social role of a person affects both the topic of a question asked and the way the question is formulated. An ideal explanation also takes the social role into account. Therefore, it would be advantageous for a robot that generates explanations to know the social role of the user. To this end, we trained a model that maps a user’s questions to her social role. The model was trained with a total of 160 training pairs $\left(q_{k,i},s_{k}\right)$, where each of the three roles $s_{k}$ was manually associated with 40 queries $q_{k,i}$ . Of these queries, 20 were sourced from real users based on the previous deployment of the robot in the retirement home, whereas 20 were linguistic variations generated using ChatGPT. Examples of the social role recognition are provided in Table 5.

**TABLE 5:** Examples of social role recognition.

Example 1. Social role: family	
*“Why did my father Alberto choose his menu?”* family, Confidence: 0.99 resident, Confidence: 0.00 technician, Confidence: 0.0	

Example 2. Social role: resident	Example 3. Social role: technician
*“Why did you remind my colleague Alberto about his physiotherapy session?”* resident, Confidence: 1.00 family, Confidence: 0.00 technician, Confidence: 0.0	*“Why do you prioritize charging over continuing your current task?”* technician, Confidence: 1.00 resident, Confidence: 0.00 family, Confidence: 0.00

In Example 1, the query “Why did my father Alberto choose his menu?” is predicted with a confidence of 0.99 to have been asked by a person with the social role family.

The remaining subsection provides a more detailed description of how the Rasa system was configured and used. In Rasa, natural language text is processed by a sequence of components in a so-called processing pipeline defined in a configuration file. The text is first split into tokens using the WhitespaceTokenizer, which creates one token for each sequence of characters separated by whitespace. The Featurizer component transforms these tokens into numerical representations (features). We utilized the RegexFeaturizer, which extracts features based on predefined regular expressions; the LexicalSyntacticFeaturizer, which extracts lexical-syntactic features using a sliding window approach; and the CountVectorsFeaturizer at both the word and character levels in the input query. At the word level, features were extracted using the bag-of-words approach capturing the frequency of occurrence of words. At the character level, features representing sub-word structures were extracted, making the model more robust to spelling variations or unseen words. Next, we utilized the DIETClassifier (Dual Intent and Entity Transformer), a component designed for joint intent classification and entity recognition, which is built on a transformer-based architecture. The DIETClassifier shares a transformer for both tasks, using a conditional random field (CRF) layer for entity recognition and a semantic vector space for intent classification (in our case, robot event and social role classification). The model optimizes by maximizing the similarity between the predicted intent vector and the correct label using dot-product loss. The outputs are the primary intent and the intent ranking with respective confidences scores. Table 4 shows various examples of the classifier’s output. Finally, the FallbackClassifier classifies a query with the intent “nlu_fallback” if the intent’s confidence score is below a defined threshold (30% in our case). The fallback intent can also be predicted when the confidence scores of the two top ranked intents are closer than the ambiguity threshold (10% in our case).

### 3.3 Retrieval of the effect and the cause

Step 2 results in a parameterized event $e_{p}$ , such as “Use_case_cognitive_started(&1),” and a named entity such as “Alberto.” In step 3, they are compared with the events in the effect column in the causal log to find rows with a matching effect β , such as “Use_case_cognitive_started(Alberto).” If more than one such row exists, the row most recently added to the causal log is chosen. In addition to the effect β , the cause α , the timestamp t , and the cause index cause_idx are retrieved from the same row. Examples of effect and cause retrieval for four queries are provided in Table 6.

**TABLE 6:** Examples of effect and cause retrieval for four queries.

Example 1. “Why did my father Alberto choose his menu?”	Example 2. “Why did my father Alberto undergo cognitive therapy?”
Primary intent: Use_case_menu_started (&1), Confidence: 0.81 Primary effect: Use_case_menu_started (&1) Final effect: Use_case_menu_started (Alberto) Full row of effect: {‘timestamp’: 1728374585; ‘cause’: Use_case_cognitive_finished ^ Person_detected (Alberto) ^ Person (Alberto).neuron = false ^ Person (Alberto).menu = “”; ‘cause_idx’: 46,25,43,27}	Primary intent: Use_case_cognitive_started (&1), Confidence: 0.87 Primary effect: Use_case_cognitive_started (&1) Final effect: Use_case_cognitive_started (Alberto) Full row of effect: {‘timestamp’: 1728374558; ‘cause’: Person_detected (Alberto) ^ Person (Alberto).neuron = true ^ Cognitive_time = true; ‘cause_idx’: 25,26,3}

Example 3. “Why did you move around the living room?”	Example 4. “Why were you interacting with Pedro?”
Primary intent: Use_case_wandering_started, Confidence: 0.87 Primary effect: Use_case_wandering_started Final effect: Use_case_wandering_started Full row of effect: {‘timestamp’: 1728383268; ‘cause’: Use_case_music_finished; ‘cause_idx’: 127}	Primary intent: Person_interacting (&1), Confidence: 0.91 Primary effect: Person_interacting (&1) Final effect: Person_interacting (Pedro) Full row of effect: No match found in the ‘effect’ column

The latest entry of an effect β is retrieved from the causal log, alongside with its cause α , timestamp t , and cause index.

### 3.4 Generation of causal explanations

An initial natural language description of the retrieved cause $\alpha =\alpha_{1},\ldots ,\alpha_{k}$ and effect β is first generated by utilizing the dictionary (see Section 2.2.2). Parameter values within parentheses, such as “Alberto,” are first substituted with placeholders (e.g., &1). The resulting $\alpha_{1},\ldots ,\alpha_{k}$ and β are then matched with the parameterized event names $e_{p}$ in the dictionary. The corresponding event descriptions are then concatenated, and all parameters are substituted back. Separators “; ” are finally added to form the initial causal explanation $exp_{\mathrm{init}}$ . Examples are shown in Table 7.

**TABLE 7:** Examples of initial causal explanations $exp_{\mathrm{init}}$ for four queries, generated by concatenating descriptions for cause and effect in the dictionary.

Example 1. “Why did my father Alberto choose his menu?”	Example 2. “Why did my father Alberto undergo cognitive therapy?”
The robot ended the cognitive therapy as it only asks a limited number of questions to avoid exhausting the user; person Alberto was detected by the robot’s facial recognition camera; person Alberto did not have the cognitive therapy activity scheduled for today or has just completed it; person had not ordered any food. Effect: the robot approached a recognized person Alberto and asked for his menu choices. Menu selection was made by touching the appropriate images on the robot’s screen	Person Alberto was detected by the robot’s facial recognition camera; person Alberto was scheduled for the cognitive therapy activity today; it was the right time for cognitive therapy. Effect: when the robot recognized Alberto, who participates in the therapy, it offered the possibility to start an activity, which is conducted using the robot’s touch screen

Example 3. “Why did you move around the living room?”	Example 4. “Why where you interacting with Pedro?”
The robot finished playing music as the hour of music therapy has ended. Effect: the robot moved to random locations in a specified area looking for opportunities to perform a use case	The robot has not seen the person

$exp_{\mathrm{init}}$ is syntactically enhanced to form a grammatically cohesive sentence that is better suited for the final refinement stage handled by the LLM. We observed that the LLM consistently produced more fluent and natural explanations when the input was a single connected sentence, rather than a sequence of disjointed clauses or bullet points. The word “because” is inserted at the beginning of the first cause to explicitly mark causality. If there are multiple causes, they are joined using commas between clauses, with the word “and” before the final cause to form a coordinated list. Additionally, occurrences of the word “Person” are removed for better readability, resulting in the syntactically enhanced explanation $exp_{\mathrm{syn}}$ . Examples are provided in Table 8.

**TABLE 8:** Examples of syntactically enhanced causal explanations $exp_{\mathrm{syn}}$ for four queries, created by adding conjunctions such as “and” or “as a result” to $exp_{\mathrm{init}}$ .

Example 1. “Why did my father Alberto choose his menu?”	Example 2. “Why did my father Alberto undergo cognitive therapy?”
Because the robot ended the cognitive therapy as it only asks a limited number of questions to avoid exhausting the user, Alberto was detected by the robot’s facial recognition camera, Alberto did not have the cognitive therapy activity scheduled for today or has just completed it, and Alberto had not ordered any food. As a result, the robot approached a recognized person, Alberto, and asked for his menu choices. Menu selection was made by touching the appropriate images on the robot’s screen	Because Alberto was detected by the robot’s facial recognition camera, Alberto was scheduled for the cognitive therapy activity today and it was the right time for cognitive therapy. As a result, when the robot recognized Alberto, who participates in the therapy, it offered the possibility to start an activity, which is conducted using the robot’s touch screen

Example 3. “Why did you move around the living room?”	Example 4. “Why where you interacting with Pedro?”
Because the robot finished playing music as the hour of music therapy had ended. As a result, the robot moved to random locations in a specified area looking for opportunities to perform a use case	The robot has not seen the person

### 3.5 Generation of final explanation

Although the generated explanations $exp_{\mathrm{syn}}$ (see Table 8) contain necessary and correct information, they are not sufficiently well expressed to be easily understood. A final adaption is, therefore, performed by utilizing the LLM Llama^1^, which allows for local and not only cloud-based processing. This enables responsive real-time interaction, suitable for on-device usage in social robotics applications. The specific model used was the Meta-Llama-3.1-8B-InstructQ4_k_M.gguf, with 8 billion parameters. It is tuned for generating instructional and conversational responses, allowing it to respond accurately and naturally to user queries.

Section 4 describes how different combinations of the factors $exp_{\mathrm{syn}}$ , s , and q were evaluated for the generation of the final explanation $exp_{\mathrm{final}}$ . For the combination with all factors, Llama was called with the following prompt: “You are a social assistive robot. According to the role [s] of the target that you are answering and the question asked [q] . Compress the answer with the meaningful information: $\left[exp_{\mathrm{syn}}\right]$ .” For other combinations of factors, the prompt was adjusted correspondingly.

## 4 Evaluation

The proposed methodology was implemented on a computer and evaluated using three experiments. Before providing detailed descriptions of these experiments, we provide a summary of the design and results of the experiments.

Experiment I assessed the quality of various system-generated explanations in terms of how well they are tailored to a specific social role. The system-generated explanations were assessed by experts and by 30 recruited participants filling out questionnaires. The vast majority of the participants were staff at the Electronic Technology Department at the University of Malaga, with some technical knowledge of programming and physical robotic systems. The results established that the most preferred explanations were generated by including the original query *q* and the social role *s* in the LLM prompt used to refine $exp_{\mathrm{syn}}$ to the final explanation $exp_{\mathrm{final}}$ (step 6 in Figure 1).

Experiment II investigated how well the system inferred the social role, given a natural language query. For this, all queries used in Experiment I were tested, and in addition, 30 survey participants generated queries to an imagined robot, assuming one of the social roles of family member, resident, and technician. As summarized in Table 10, the accuracy for recognition of the three social roles varied between 83% and 90%.

Experiment III evaluated the system-generated explanations by calculating the cosine similarity between human-generated explanations, serving as ground truth, and system-generated explanations. As reported in Table 11, the cosine similarities were maximized for system-generated explanations aimed for the same social role as the human-generated explanations. For all three roles, these similarity scores were higher than 86%. The three conducted experiments are described in detail in the following subsections.

### 4.1 Experiment I

The first experiment aimed at investigating the effect of providing various types of information to the LLM for the generation of explanations. In particular, for each original query, four use cases were considered: *Why did Alberto choose from the menu today?*, *Why did you give cognitive therapy to Alberto?*, *Why did you detect Jose?*, and *Why are you starting to move around*; corresponding modified questions, tailored to one of the social roles of family member, resident, and technician, were then generated. For each such modified question, four combinations of $exp_{\mathrm{syn}}$ , s , and q were used to formulate four LLM prompts, as described in Section 3.5. The combinations, denoted C1−C4 , were defined as follows:

C1 : $exp_{\mathrm{syn}}$ .

C2 : $exp_{\mathrm{syn}}$ and q .

C3 : $exp_{\mathrm{syn}}$ and s .

C4 : $exp_{\mathrm{syn}}$ , s , and q .

The quality and adequacy of the output of the final explanation $exp_{\mathrm{final}}$ for $C_{1}-C_{4}$ was then assessed by 30 test participants. In particular, they were asked to mark which one of the explanations generated through the four combinations C1−C4 they preferred, considering semantic adequacy, linguistic efficiency, temporal contextualization, and social role. By the term contextualization, we refer to correct identification of the cause-and-effect structures, and by the term personalization, we refer to the explanations being tailored to the social role of the human and to the linguistic subtleties in the natural language query. Note that each participant assessed the suitability of the generated questions for assumed interlocutors with different social roles. The age distribution of the participants was as follows: under 30 years (6.67%), 30–44 years (23.33%), 45–60 years (43.33%), and over 60 years (26.67%). A total of 56.67% of the participants were male and 43.33% were female participants. Details are reported in the next section, and a tabular summary of the 48 explanations preferred by the participants is provided in Section 4.1.1.

Additionally, the authors of the paper conducted an expert evaluation of the obtained final system-generated explanation regarding the identification of social roles s and events e , and also the differences between the four combinations C1−C4 of information (see details in Section 4.1.1).

For all examined cases, the social role s recognized by Rasa as described in Section 3.2 matched the social role assumed when constructing the three modified social role questions. All events e were also correctly recognized for all modified questions. As a result, the initial explanations $exp_{\mathrm{init}}$ (and thereby $exp_{\mathrm{syn}}$ ) were also the same.

In the following, all use case queries and all 48 assessed explanations are listed.

For ease of reference, each explanation is marked with a sequential number within parentheses. The percentage of test participants preferring one of the four versions C1−C4 is added after the explanation.

Example 1 System-generated explanations for use _ case _ menu.

Original query: Why did Alberto choose from the menu today?

Recognized event *e*: Use_case_menu_started (Alberto).

$exp_{\mathrm{syn}}$ : Because the robot ended the cognitive therapy as it only asks a limited number of questions to avoid exhausting the user, Alberto was detected by the robot’s facial recognition camera, Alberto did not have the cognitive therapy activity scheduled for today or has just completed it, and Alberto had not ordered any food. As a result, the robot approached a recognized person Alberto and asked for his menu choices. Menu selection was made by touching appropriate images on the robot’s screen.

#### 4.1.1 Social role: family

Modified social role question *q*: Why did my father Alberto choose his menu today?

Generated final explanations $exp_{\mathrm{final}}$ :

C1: (1) I approach Alberto because I detected him through my camera, and as he does not have a scheduled therapy session or a food order, I am here to help him choose his meal. 0%
C2: (2) I approached Alberto because he was recognized by my facial recognition camera, and as he did not have a scheduled cognitive therapy session today, I asked him to order his meal by selecting from the menu on my screen. 0%
C3: (3) Good morning, Alberto! I am here to help. I noticed you are here, so I shall ask, would you like to order some lunch today? 0%
C4: (4) I helped your father order the menu because I finished his therapy session and saw that he had not eaten yet, so I offered him the menu on my screen. 100%

#### 4.1.2 Social role: resident

Modified social role question *q*: I am Alberto, why did you ask me to select my menu?

Generated final explanations $exp_{\mathrm{final}}$ :

C1: (5) I approach Alberto because I detected him through my camera and he was not scheduled for therapy today, and he has not ordered food, so I am offering him the menu to make a selection. 0%
C2: (6) I asked you to select your menu, Alberto, because I detected you with my camera and saw you were not in therapy today, and you have not ordered food yet, so I wanted to help you choose your meal. 0%
C3: (7) I am here to assist you, Alberto. I noticed you were here, so I shall show you the menu for today. Can you please touch the images on my screen to choose what you would like to eat? 26.67%
C4: (8) I asked you to select your menu because I recognized you, saw you had finished therapy, and knew you had not ordered yet, so I helped you choose your meal. 73.33%

#### 4.1.3 Social role: technician

Modified social role question *q*: Why did the system ask Alberto to choose from the menu today?

Generated final explanations $exp_{\mathrm{final}}$ :

C1: (9) I approach Alberto because I detected him with my camera, and as he does not have a scheduled therapy session or a food order, I am offering him the menu to select his meal. 6.67%
C2: (10) I ordered the menu to Alberto because I detected him with my camera, he did not have a scheduled therapy session today and had not ordered food yet, so I approached him to offer the menu, and he can select his choices by touching the screen. 23.33%
C3: (11) I am approaching Alberto because I detected him with my camera and he does not have a scheduled therapy session or a food order, so I am offering him the menu to make a selection. 0%
C4: (12) I selected the menu for Alberto because our system detected him through facial recognition and determined he was done with therapy and had not ordered food yet, so I prompted him to choose from the menu on the screen. 70%

Example 2 System-generated explanations for use _ case _ cognitive therapy

Original query: Why did you give cognitive therapy to Alberto?

Recognized event *e*: Use_case_cognitive_started (Alberto).

$exp_{\mathrm{syn}}$ : Because Alberto was detected by the robot’s facial recognition camera, Alberto was scheduled for the cognitive therapy activity today, and it was the right time for cognitive therapy. As a result, when the robot recognized Alberto who participates in the therapy, it offered the possibility to start an activity, which is conducted using the robot’s touch screen.

#### 4.1.4 Social role: family

Modified social role question *q*: Why did you give cognitive therapy to my father Alberto?

Generated final explanations $exp_{\mathrm{final}}$ :

C1: (13) I am starting the cognitive therapy activity with Alberto because I detected him through my camera and it was his scheduled time today. 0%
C2: (14) I gave cognitive therapy to your father Alberto because I detected him through my camera and it was his scheduled therapy time today. 0%
C3: (15) I am here to help Alberto with his cognitive therapy session, which is scheduled for today. 16.67%
C4: (16) I gave your father cognitive therapy because I recognized him and knew he had a scheduled session today, so I offered him the activity on my screen. 83.33%

#### 4.1.5 Social role: resident

Modified social role question *q*: I am Alberto, why did you ask me to start the cognitive session?

Generated final explanations $exp_{\mathrm{final}}$ :

C1: (17) I am recognizing Alberto through the camera, and as he is scheduled for cognitive therapy today, I am offering him the chance to start the activity on my touch screen. 3.33%
C2: (18) I asked you some questions, Alberto, because I detected you through my camera and it was your scheduled therapy time today. 6.67%
C3: (19) I am here to help you, Alberto, because it is time for your cognitive therapy session today, and I have been programmed to assist you with it. 26.67%
C4: (20) I asked you questions because I recognized you and knew it was time for your therapy session, so I offered you the activity on my screen. 63.33%

#### 4.1.6 Social role: technician

Modified social role question *q*: Why did you perform the cognitive therapy use case to Alberto?

Generated final explanations $exp_{\mathrm{final}}$ :

C1: (21) I am starting the cognitive therapy activity with Alberto because I have recognized him through my camera and it is his scheduled time. 3.33%
C2: (22) I performed the cognitive therapy use case with Alberto because I detected him through my facial recognition camera, and he had a scheduled therapy session today, which is the optimal time for it. 13.33%
C3: (23) I am recognizing Alberto through the facial recognition camera, and as he is scheduled for cognitive therapy today, I am offering him the opportunity to start the activity on my touch screen. 26.67%
C4: (24) I performed the cognitive therapy use case with Alberto because our system detected him through facial recognition and confirmed he had a scheduled therapy session today, so I initiated the activity on my screen. 56.67%

Example 3 System-generated explanations for use _ case _ person detection

Original query: Why did you detect Jose?

Recognized event *e*: Person_detected (Jose).

$exp_{\mathrm{syn}}$ : Because Jose was sitting or standing in front of the robot, the light level in the room was sufficient for the robot’s camera, and Jose’s face is stored in the robot’s database. As a result, Jose was detected through the robot’s facial recognition camera.

#### 4.1.7 Social role: family

Modified social role question *q*: Why did you detect my father Jose?

Generated final explanations $exp_{\mathrm{final}}$ :

C1: (25) I detected Jose because he was in my line of sight, the room was well-lit, and his face is stored in my database, allowing my facial recognition camera to identify him. 0%
C2: (26) I detected your father Jose because he was in front of me, the room was well-lit, and his face was in my database, allowing my facial recognition camera to identify him. 0%
C3: (27) Ah, Jose is here! I have recognized him through my facial recognition system, which uses the camera to detect him when he is in front of me and the lighting is good, and as his face is stored in my database. 20%
C4: (28) I detected your father Jose because he was in front of me, the room had sufficient lighting, and his face is stored in my database, allowing me to recognize him through my facial recognition camera. 80%

#### 4.1.8 Social role: resident

Modified social role question *q*: I am Jose, why did you detect me?

Generated final explanations $exp_{\mathrm{final}}$ :

C1: (29) I detected Jose because he was in my line of sight, the room was well-lit, and his face is stored in my database, allowing my facial recognition camera to identify him. 10%
C2: (30) I detected you, Jose, because you are in front of me, the room is well-lit, and your face is stored in my database, allowing my facial recognition camera to identify you. 0%
C3: (31) I am glad I was able to catch your attention, Jose! My system recognized you because you are right in front of me, the lighting is good, and I have your face stored in my memory. It is nice to see you! 30%
C4: (32) I detected you, Jose, because you were in front of me, the room had good lighting, and your face is stored in my database, which allowed me to recognize you through my facial recognition camera. 60%

#### 4.1.9 Social role: technician

Modified social role question *q*: Why did your recognition system detect Jose?

Generated final explanations $exp_{\mathrm{final}}$ :

C1: (33) I detected Jose because he was in my line of sight, the room was well-lit, and his face is stored in my database, allowing my facial recognition camera to identify him. 26.67%
C2: (34) My recognition system detected Jose because he was in my direct view, the room had sufficient lighting, and his face is stored, allowing facial recognition to identify him. 13.33%
C3: (35) System check confirms that Jose’s face was detected by the facial recognition camera due to optimal lighting conditions and his stored profile in the database, indicating a successful recognition event. 0%
C4: (36) Our recognition system detected Jose because he was in direct view of the camera, the room’s lighting was adequate for the camera, and his face is stored in our database, which enabled our facial recognition camera to identify him. 60%

Example 4 System-generated explanations for use _ case _ wandering

Original query: Why are you starting to move around?

Recognized event *e*: Use_case_wandering_started.

$exp_{\mathrm{syn}}$ : Because the robot finished the request, having already notified the therapist of the user’s need. As a result, the robot moved to random locations in a specified area looking for opportunities to perform a use case.

#### 4.1.10 Social role: family

Modified social role question *q*: Why are you starting to move around my father’s room?

Generated final explanations $exp_{\mathrm{final}}$ :

C1: (37) Notified therapist, moving to random locations to perform use case. 10%
C2: (38) I am moving around your father’s room because I have completed the task of notifying the therapist of his need, and now I am exploring the area to identify opportunities to assist him. 3.33%
C3: (39) I have completed the task and notified the therapist. Now, I am roaming the area, looking for opportunities to engage with the residents and perform a use case, perhaps assisting with medication reminders, providing companionship, or helping with daily tasks. 0%
C4: (40) I am moving around your father’s room because I have finished assisting with his request, and now I am exploring the area to see if there are any other ways I can help him, perhaps by reminding him of his medication schedule or helping him find something he needs. 86.67%

#### 4.1.11 Social role: resident

Modified social role question *q*: Why are you starting to move around?

Generated final explanations $exp_{\mathrm{final}}$ :

C1: (41) Notified therapist, moving to random locations to perform use case. 0%
C2: (42) I have completed the task of notifying the therapist of the user’s need. Now, I am moving to random locations in the specified area to identify opportunities to perform a use case and assist the residents. 0%
C3: (43) Hello! I am glad to see you are doing well today. I have finished helping with your request, and I am now exploring the area to see if I can assist you with anything else, perhaps reminding you of your medication schedule or helping you find something in the activity room? 46.67%
C4: (44) I am moving around because I have completed my current task of notifying the therapist about the user’s need, so I am now exploring the area to identify new opportunities to assist and perform various use cases. 53.33%

#### 4.1.12 Social role: technician

Modified social role question *q*: Why did your system decide to start moving?

Generated final explanations $exp_{\mathrm{final}}$ :

C1: (45) Notified therapist, moving to random locations to perform use case. 16.67%
C2: (46) I started moving because I completed the task of notifying the therapist of the user’s need, so I am now exploring the area to identify opportunities to assist the residents. 6.67%
C3: (47) System check: task completed, user’s need notified to the therapist. Now, I am roaming the designated area, scanning for opportunities to engage in use cases, such as medication reminders, social interaction, or task assistance, to ensure optimal support for the residents. 0%
C4: (48) My system decided to start moving because I have completed the current task of notifying the therapist about the user’s need, and now I am entering autonomous mode to proactively seek out new opportunities to assist and perform various use cases in the specified area. 76.67%

#### 4.2.1 Analysis of the results from experiment I

##### 4.2.1.1 Expert evaluation

The effect of including varying amount of information (C1–C4) in the generation of explanations was assessed by the authors and by survey participants. Comparing explanations based on C2 and C4 (both including the query *q*) with C1 and C3 (neither including the query *q*), it becomes clear that *q* contains important information that is not reflected in the event *e* extracted from *q*. This additional information allows for explanations that, for example, use the correct and specific pronouns, such as “your menu” (6) and “your father” (14, 26, and 38). Omitting this information sometimes leads to explanations with incorrect pronouns, such as “Good morning Alberto … ” (3) although the query states “Why did my father Alberto … ,” “I’m recognizing Alberto … ” (17) although the query states “I am Alberto … ,” and “Ah, Jose is here!…” (27) although the query states “Why did you detect my father … ”

Adding the social role *s* of the user (C3 and C4) clearly adapts the language of the explanations to the assumed social context. Some examples for the social roles family and resident are as follows: “I’m here to help … ” (15, 19), “I noticed you’re here … ” (3, 7), and “I recognized you … ” (20). Some examples for the social role technician are as follows: “Our system detected him through facial recognition … ” (24), “…touch screen” (23), “…initiated the activity … ” (24), and “entering autonomous mode … ” (48).

##### 4.2.1.2 Participant assessment

Our qualitative assessment above matched the survey participants’ preferences reported in the questionnaires. The percentages reported (see Table 9 for an overview) indicate that C4 (including both *s* and *q*) is preferred by a majority of the participants for all investigated cases.

**TABLE 9:** Overview percentage of the preferred system-generated explanations.

	C1: $exp_{\mathrm{syn}}$	C2: $exp_{\mathrm{syn}}$ + q	C3: $exp_{\mathrm{syn}}$ + s	C4: $exp_{\mathrm{syn}}$ + s + q
Use case menu				
Family member	0%	0%	0%	100%
Resident	0%	0%	26.67%	73.33%
Technician	6.67%	23.33%	0%	70%
Use case cognitive therapy				
Family member	0%	0%	16.67%	83.33%
Resident	3.33%	6.67%	26.67%	63.33%
Technician	3.33%	13.33%	26.67%	56.67%
Use case person interaction				
Family member	0%	0%	20%	80%
Resident	10%	0%	30%	60%
Technician	26.67%	13.33%	0%	60%
Use case wandering				
Family member	10%	3.33%	0%	86.67%
Resident	0%	0%	46.67%	53.33%
Technician	16.67%	6.67%	0%	76.67%
Total	6.39%	5.56%	16.11%	71.94%

C1−C4 denote different combinations of syntactically enhanced explanations $exp_{\mathrm{syn}}$ , social roles s , and original queries q that are used to construct the prompt to the LLM for generation of the final explanation $exp_{\mathrm{final}}$ .

Pairwise chi-square tests were conducted to confirm that the number of votes for C4 was larger than that for each of C1, C2, and C3.

These tests were conducted on the data aggregated over all user cases and social roles, with the null hypotheses that the preference for C4 is the same as that for C1, C2, and C3.

In all cases, the null hypothesis could be rejected with p-values ≪0.0001 . Although responses were not strictly independent as each participant answered multiple questions, the very low p-values strongly suggest that C4 is the most preferred option.

It should be noted that this conclusion is based on the aggregated data and that the situation for individual use cases/social roles may differ. However, such an analysis would require a larger study for statistical significance.

Hence, we conclude that generated explanations benefit from the extra information provided by both original query *q* and social role *s*. Query *q* contains additional information that is not included in recognized event *e*, and *s* enables adaption of both language and content to fit the social context and the user’s specific need for information.

C3 (including the social role s but not the query q ) is the second most preferred option, indicating that social context plays an important role for the generation of explanations. C1 , that is, including only cause-and-effect information, is preferred roughly as often as C2 , in which the query q is added.

### 4.2 Experiment II

The trained Rasa intent recognizer successfully inferred the social roles associated with all questions in Experiment I. To further evaluate this ability, an additional experiment was conducted with 30 participants (same as in Experiment I). The participants were asked to assume the three social roles and formulate open questions to a hypothetical robot, for example,

• Family: Why did not you remind my father about the physiotherapy appointment earlier?

• Resident: Why did you ask John what he wants to eat before you asked me?

• Technician: Why did the system decide to give the menu to Alberto?

For each of the resulting 90 questions, the role was inferred by Rasa and compared with the actual role assumed by the participant formulating the question. The overall accuracy is summarized in Table 10. For example, for 27 of the 30 questions asked by participants assuming the family role, the role was correctly inferred. Incorrect inferences were mainly related to the distinction between the resident and technician, particularly for questions of a technical informative nature asked in a colloquial tone, such as “Why did the system decide to give the menu to Alberto?”. Averaged over all social roles, the accuracy was 84%(76/90) .

**TABLE 10:** Social role recognition performance.

Role	Accuracy
Family	90.00% (27/30)
Resident	80.00% (24/30)
Technician	83.33% (25/30)

### 4.3 Experiment III

To evaluate how well the system-generated explanations matched human-generated explanations for given social roles, the following experiment was conducted. For each of the questions in the use case examples 1–4 (see Section 4.1) and for each social role, a human-generated explanation was generated by one of the authors. For example, for “Why did Alberto choose from the menu today?” (example 1), the following explanations were generated:

• Family: Your father chose the menu today because he had finished therapy and did not have lunch yet.

• Resident: You selected the menu because you had completed your therapy and not yet ordered it.

• Technician: I selected the menu case for him because I determined he was done with therapy and had not ordered food yet.

For each question and social role, a system-generated final explanation $exp_{\mathrm{final}}$ was also created. The cosine similarity between the embedding of the final explanation $exp_{\mathrm{final}}$ and the embedding of the corresponding human-generated explanation was then computed and assigned as the quality q of the final explanation $exp_{\mathrm{final}}$ . The procedure was repeated for all three social roles and the four questions in examples 1–4. Average q values are presented in Table 11. The maximum value for each row lies on the diagonal in the table, which confirms that the system adapts the explanations well to the targeted social role.

**TABLE 11:** Cosine similarity between system-generated explanations and human-generated reference explanations for different targeted social roles.

System-generated explanation	Human-generated explanation		
	Family, %	Resident, %	Technician, %
Family	90.17	76.45	78.88
Resident	76.10	86.41	79.31
Technician	77.27	77.13	88.71

## 5 Discussion and limitations

We examined how integrating social roles, user queries, and cause-and-effect structures influences the generation and perception of causal explanations. The presented framework combines manual control for factual causal correctness (via the causal log) and flexibility and variety in linguistic expression (via LLMs).

As mentioned in Section 2.1, some related earlier studies argue for the importance of adapting to the abilities and needs of the inquirer and bridging the mental gap to enable comprehension. Our proposed methodology addresses this by incorporating the social role and the verbally expressed query that potentially encodes beliefs, desires, and other relevant parts of a mental model of the inquirer.

Although the proposed framework shows promise in generating personalized explanations, several challenges remain. The ethical implications extend beyond transparency, touching on issues of integrity, fairness, bias, and the risk of manipulation. Furthermore, explanations must not exploit user vulnerabilities or reinforce harmful stereotypes. In particular, in elder care settings, sensitive personal data, such as levels of cognitive or physical fitness, should be handled with utmost integrity and safety and not be verbalized by the robot when interacting with a resident. Verbally communicating sensitive personal data, in particular in common rooms with other bystanders, may make a resident in an elder care home feel embarrassed, shameful, or stereotyped (Bensch et al., 2023). Trust calibration also remains a central concern; explanations should align user expectations with the robot’s actual capabilities to avoid over-reliance or distrust.

The proposed identification of the social role of the enquirer calls for ethical considerations related to personal integrity. Visiting family members, for example, may or may not want to be identified as such. To ensure that sensitive personal information is not shared with outsiders, electronic ID cards or badges could be a crucial complement. Another important issue that would have to be considered in a real implementation is adaptation to different languages and cultures. Social roles, linguistic expressions, and expectations connected to explanations can vary significantly between cultures and languages. For example, what is considered an appropriate or respectful tone in one culture might be perceived as overly formal or informal in another. Similarly, the interpretation of causal responsibility can differ in different contexts.

In the evaluation, the survey participants assessed the system-generated explanations in terms of their adequacy for an assumed social role. This should be tested and evaluated with users who actually have the social role of, for example, medical staff or family member.

The presented solution offers a robust starting point, combining manual control for factual causal correctness and flexibility of LLMs, but its reliance on specific pretrained models and predefined user interaction modes may limit its flexibility in unstructured environments. Additionally, the computational requirements of LLM-based explanation generation may pose challenges in resource-constrained scenarios. Addressing these limitations will require both algorithmic innovation and hardware optimization.

Additionally, even though the causal log entries are extracted from actual robot sensors, much manual inspection is still required, and the dictionary that describes the causal log entries in natural language has to be created manually. In this paper, we use the causal log to extract only the most recent event for which an explanation was asked; however, the tabular form and indexing allow the causal log to be used for longer causal chains.

## 6 Conclusion and future work

We introduced a framework and methodology that enable robots to generate personalized causal explanations of robot events. By representing robot events as cause–effect structures in a causal log that represents the robot’s episodic memory, causal correctness is preserved and causal data are transparent. Using machine learning, the human’s social role is identified and is, together with the causal data and the natural language query, given to an LLM that then generates linguistically varied causal explanations. We evaluated our approach with 30 participants, who assessed explanations that combined cause–effect reasoning, the social role, and the natural language query in different ways. The results show that 72% of the survey participants preferred explanations that integrate all three factors. The second most preferred option was explanations based on the cause-and-effect structures and the social role (see Table 9 for details).

Future work could investigate whether preferences depend on the social role and specific use cases.

Further evaluations show that the social role of the enquirer was inferred from the query with an accuracy of 79% (see Table 10). It was also shown that system-generated explanations tailored to a specific social role have the highest semantic similarity to human-generated explanations aimed for the same social role (see Table 11). This indicates that the adaption of explanations to the social role of the enquirer works as intended.

Overall, the presented solution addresses a critical need for personalized and linguistically varied explanations. We believe that such functionality increases user engagement, as shown in an earlier study where users of a social assistive robot used at a retirement home in Malaga, Spain, remarked on the linguistic monotony of the robot.

The presented methodology has been integrated into a complex robotic cognitive architecture and implemented on a social robot (Galeas et al., 2025). As a planned next step, the operation will be evaluated in a retirement home with real users. This will involve adapting the software to work seamlessly with robotic hardware, including sensors, actuators, and real-time processing capabilities. Deploying the system in this dynamic environments will provide valuable insights into robustness, usability, and scalability under real-world conditions. Future work on the theoretical aspects will investigate explanations that include indirect causes. As discussed in Section 2.2.4, the decision on which direct and indirect causes to include is a trade-off. While explanations must be accessible and intuitive, they should also accurately reflect the underlying decision-making process. Over-simplification risks reduce the fidelity of explanations, potentially leading to user misconceptions, whereas overly complex explanations may overwhelm nonexpert users. This balance becomes particularly important in safety-critical domains, such as healthcare and eldercare, where misunderstandings can have significant consequences. Striking this balance will require both algorithm development and iterative testing with diverse user groups to develop optimal explanation strategies for various application domains. Another possible extension is to investigate how markers such as intonation, facial expression, dress code, and age could be used to further improve the personalization of causal explanations.

Through these efforts, we aim to advance the field of explainable or understandable robots, bringing us closer to realizing the vision of socially intelligent robots that seamlessly integrate into our daily lives.

## Data Availability

The raw data supporting the conclusions of this article will be made available by the authors, without undue reservation.

## Footnotes

1
https://github.com/ggerganov/11ama.cpp
